# Supplementary material for: Increased Circulating Epithelial Tumor Cells (CETC/CTC) over the Course of Adjuvant Radiotherapy Is a Predictor of Less Favorable Outcome in Patients with Early-Stage Breast Cancer
Source: Curr Oncol. 2022 Dec 24;30(1):261–73. doi: 10.3390/curroncol30010021 (PMC9857667; doi:10.3390/curroncol30010021)
Supplement: Supplementary file 1 [file curroncol-30-00021-s001.zip › curroncol-2042530-supplementary.pdf]

Supplementary Material

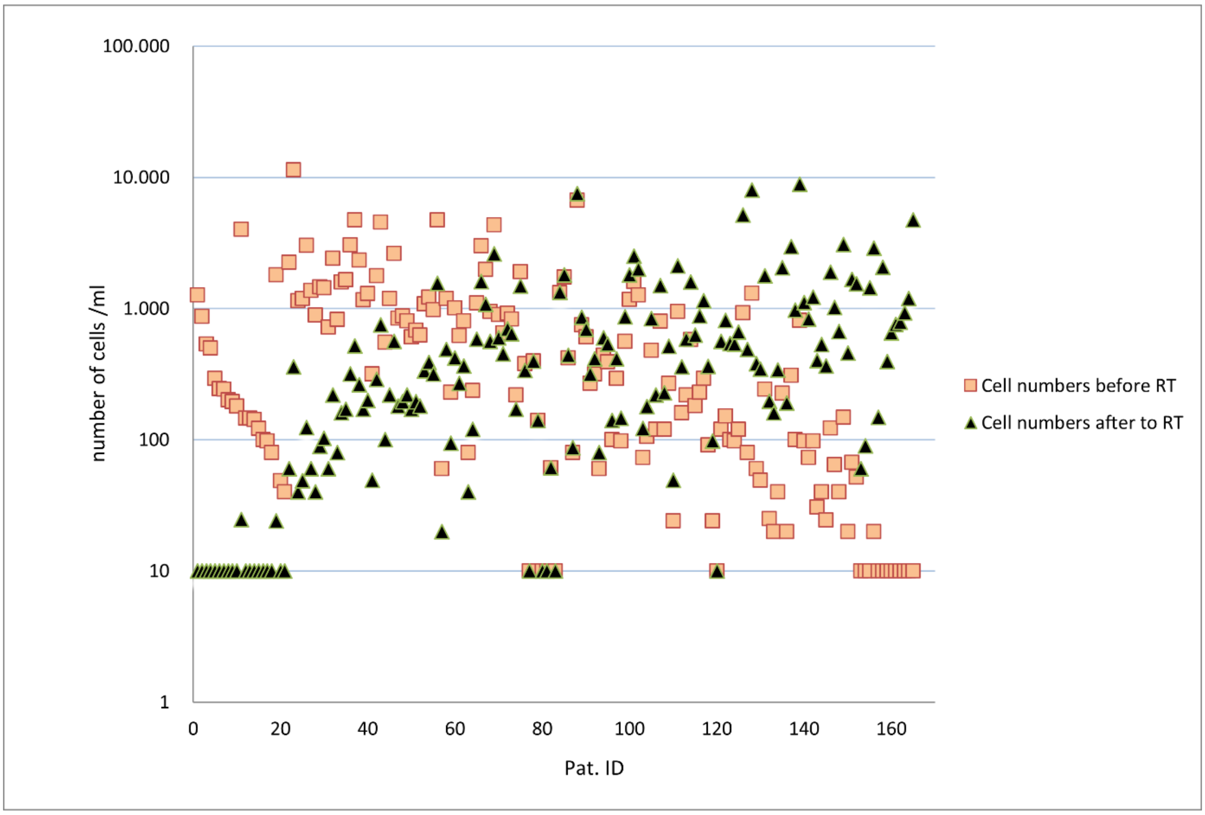

Figure S1. Absolute cell numbers before and after RT, sorted by quotient.

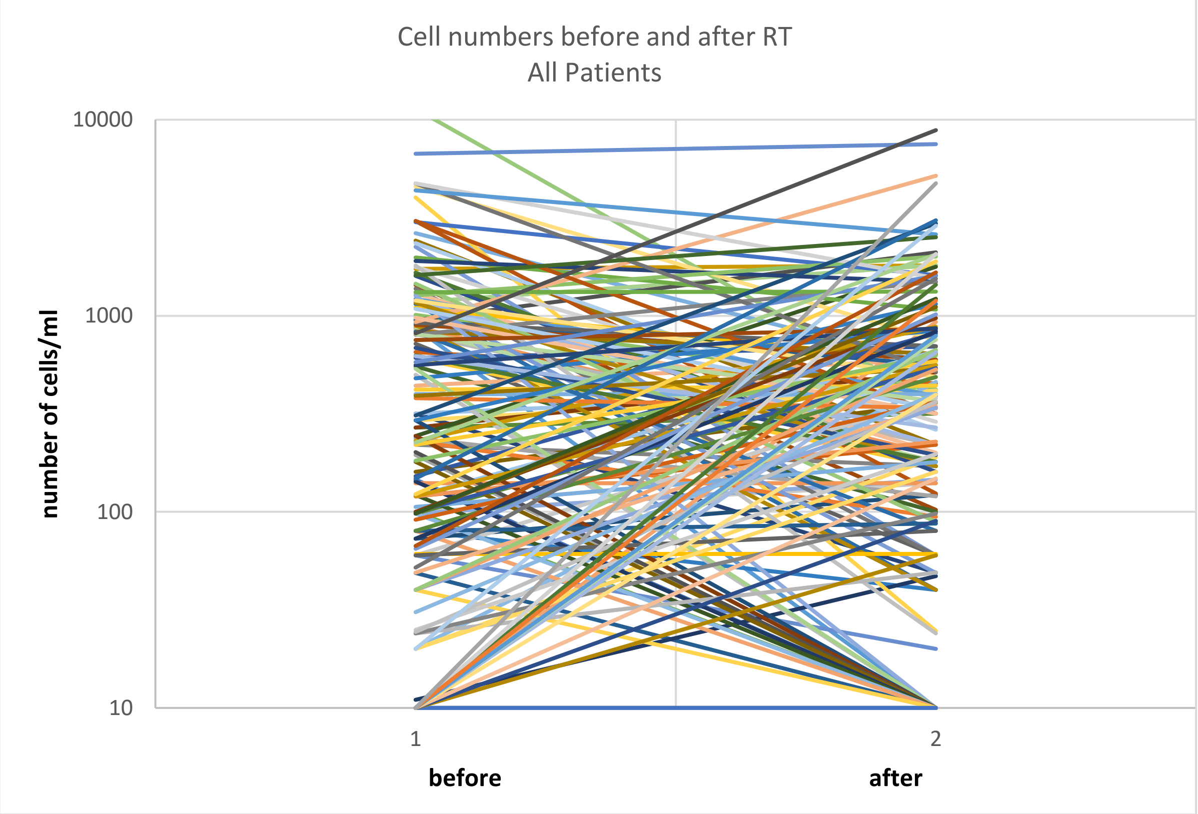

Figure S2A. Course of absolute cell counts before and after RT for all 165 patients.

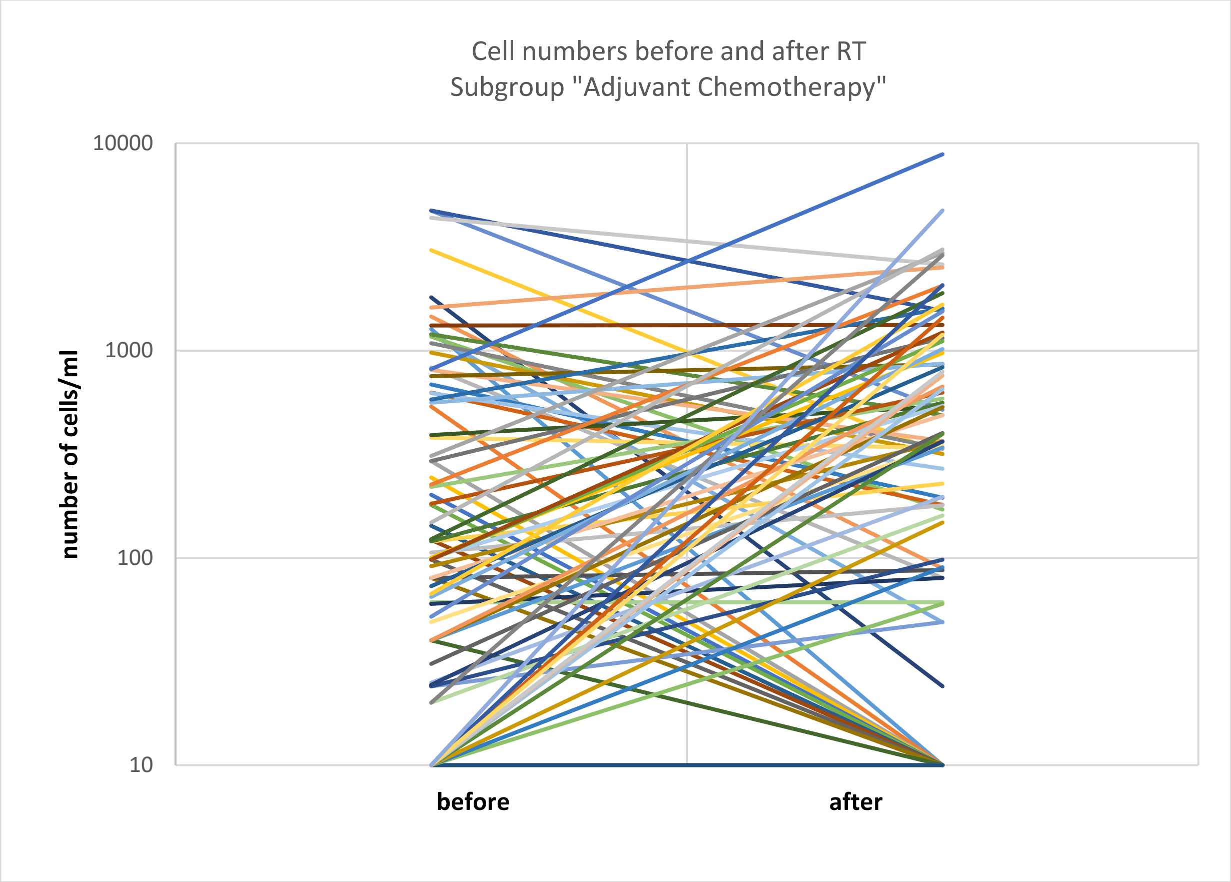

Figure S2B. Course of absolute cell counts before and after RT of patients who received adjuvant chemotherapy.

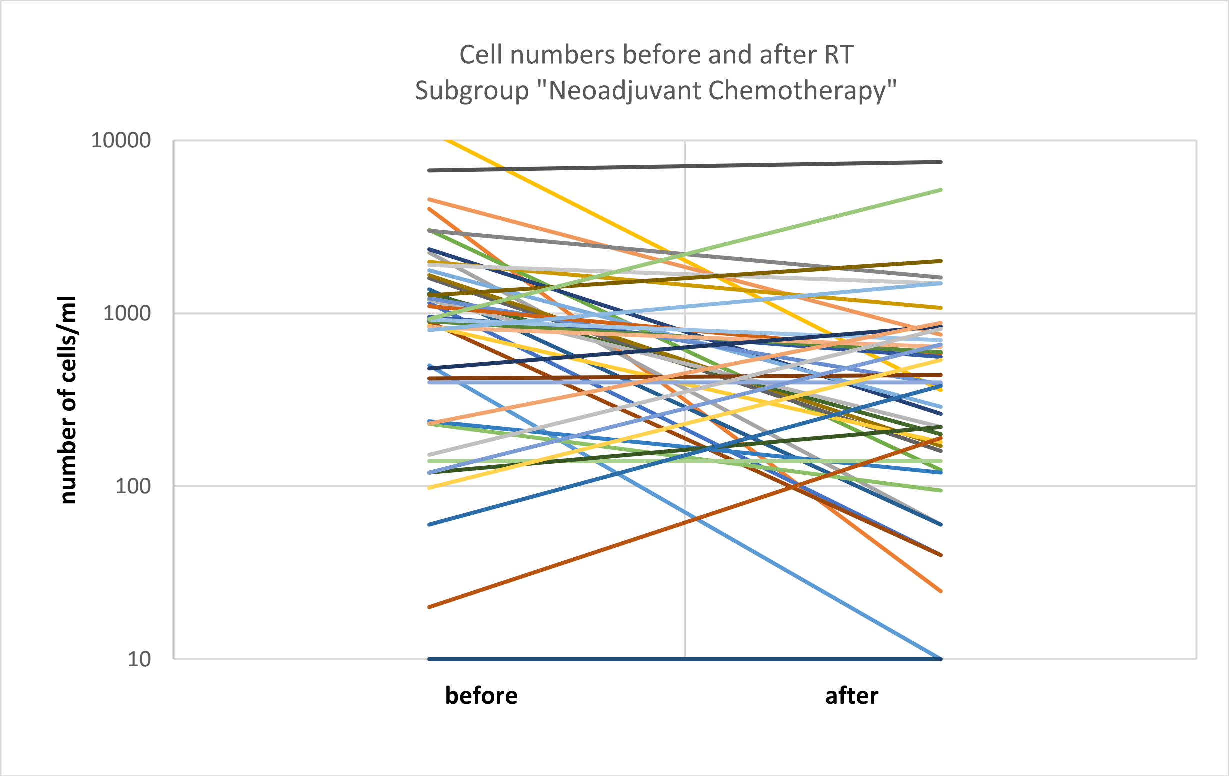

Figure S2C. Course of absolute cell counts before and after RT of patients who received neoadjuvant chemotherapy.

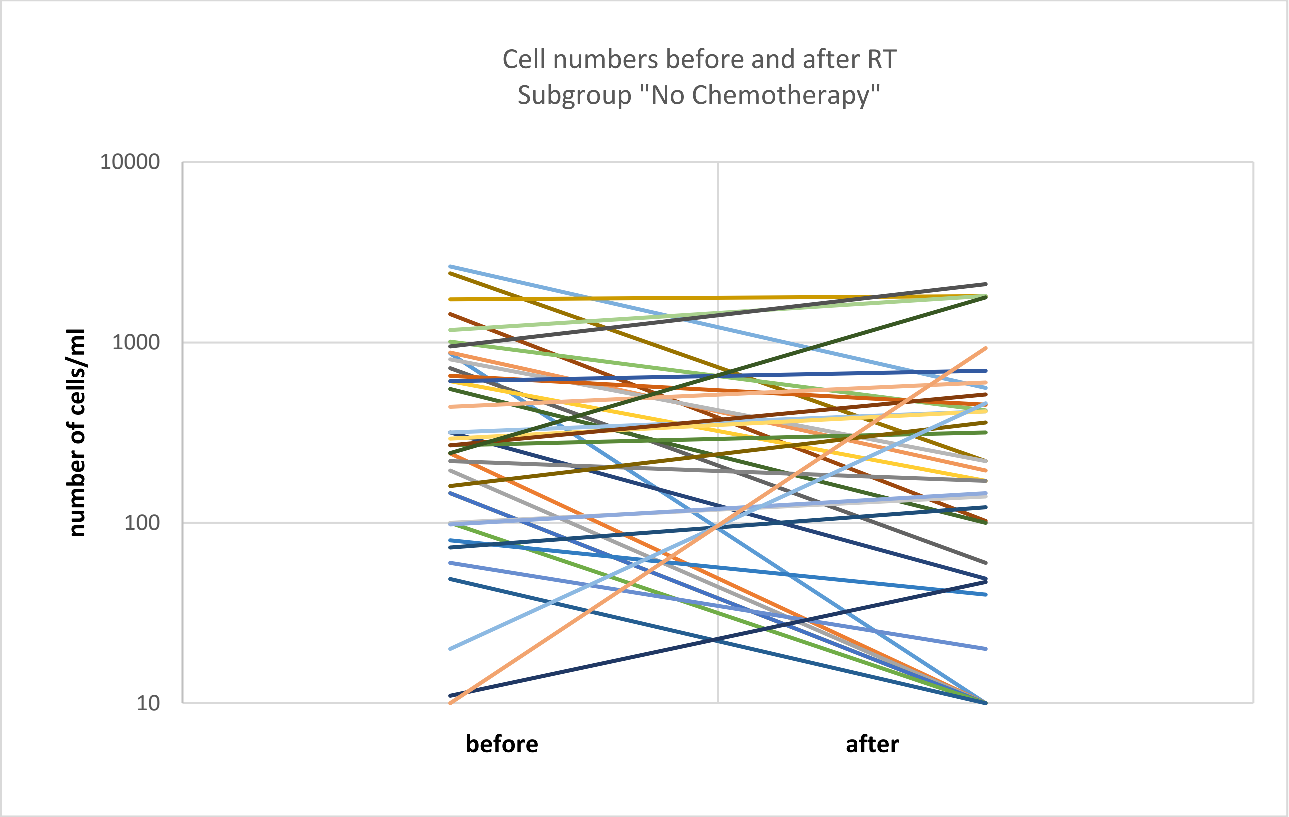

Figure S2D. Course of absolute cell counts before and after RT of patients who did not received chemotherapy.
